# Prevalence of molar incisor hypomineralization and regional differences throughout Japan

**DOI:** 10.1186/s12199-018-0748-6

**Published:** 2018-10-31

**Authors:** Masato Saitoh, Yuki Nakamura, Mika Hanasaki, Issei Saitoh, Yuji Murai, Yoshihito Kurashige, Satoshi Fukumoto, Yukiko Asaka, Masaaki Yamada, Michikazu Sekine, Haruaki Hayasaki, Shigenari Kimoto

**Affiliations:** 1 Division of Pediatric Dentistry, School of Dentistry, Health Sciences University of Hokkaido, 1757 Kanazawa, Tobetsu, Ishikari, Hokkaido 061-0293 Japan; 2 Division of Pediatric Dentistry, Graduate School of Medical and Dental Sciences, Niigata University, Niigata, 951-8514 Japan; 3 Division of Pediatric Dentistry, Department of Oral Health and Development Sciences, Tohoku University Graduate School of Dentistry, Sendai, 980-8575 Japan; 4 Department of Epidemiology and Health Policy, University of Toyama Graduate School of Medicine and Pharmaceutical Sciences, Toyama, 930-0194 Japan; 5 Department of Dentistry for Growth and Development of Oral Function Pediatric Dentistry, Kanagawa Dental University Graduate School of Dentistry, Yokosuka, 238-8580 Japan; 6 Committee of Clinical Research Promotion, Japanese Society of Pediatric Dentistry, Tokyo, 170-0003 Japan

**Keywords:** Molar incisor hypomineralization, Prevalence rates, Regional differences

## Abstract

**Background:**

Molar incisor hypomineralization (MIH) frequently occurs in children worldwide. However, MIH prevalence throughout Japan has not yet been investigated. The purpose of this study was to clarify MIH prevalence rates and to consider potential regional differences throughout Japan.

**Methods:**

A total of 4496 children aged 7–9 years throughout Japan were evaluated in this study. MIH prevalence rates among children were evaluated in eight regions throughout Japan. A child’s residence was defined as the mother’s residence during pregnancy. The localization of demarcated opacities and enamel breakdown was recorded on a standard code form using a guided record chart. Logistic regression analysis was used to evaluate whether MIH prevalence rates differed among age groups, sex, and regions.

**Results:**

The overall prevalence of MIH in Japan was 19.8%. The prevalence of MIH was 14.0% in the Hokkaido region, 11.7% in the Tohoku region, 18.5% in the Kanto Shin-Etsu region, 19.3% in the Tokai Hokuriku region, 22.3% in the Kinki region, 19.8% in the Chugoku region, 28.1% in the Shikoku region, and 25.3% in the Kyushu region. These regional differences were statistically significant. Moreover, MIH prevalence rates decreased with age. No significant sex differences in MIH prevalence rates were demonstrated.

**Conclusions:**

To our knowledge, this is the first MIH study carried out in several regions throughout Japan. Regional differences existed in MIH prevalence rates; particularly, MIH occurred more frequently in children residing in southwestern areas than those in northeastern areas of Japan.

## Highlights

- The overall prevalence of MIH in Japan was 19.8%.
- MIH occurred more frequently in southwestern areas than in northeastern areas of Japan.
- The prevalence of MIH decreased with increasing age.

## Background

Enamel hypomineralization can lead to caries development, occlusion abnormality, and esthetic issues; thus, it is an important problem in pediatric dentistry [1]. Molar incisor hypomineralization (MIH) is defined as the hypomineralization of systemic origin of one or more of the four permanent first molars and is frequently associated with affected incisors [2]. MIH often occurs in children worldwide. Large variations in MIH prevalence have been reported. Most MIH prevalence studies have been conducted in European countries, and MIH prevalence rates between 3.6 and 25.0% were reported [3–5]. The only study of MIH prevalence in Japan included 2121 elementary school children in Chiba, a suburb in Tokyo [6]. However, the examination of MIH prevalence throughout Japan has not been investigated.

Etiologies of MIH include systemic conditions at around the time of birth. Reports have suggested many possible causes during childhood such as asthma, pneumonia, respiratory infections, otitis media, tonsillitis, and chicken pox, and early use of amoxicillin and dioxins in mother’s milk [7]. However, the exact etiology of MIH remains unclear. Because MIH is associated with a systemic disturbance that occurs during the development of the permanent first molars and incisors, one leading hypothesis is that MIH is influenced by environmental factors during enamel development of these teeth.

Clinical management of MIH is difficult due to the rapid development of dental caries, limited cooperation of children, and repeated breakdown of restorations [8]. Moreover, MIH often occurs in areas with low caries prevalence [9]. Therefore, it is essential to recognize the existing state of MIH throughout Japan. The purpose of this study was to clarify the MIH prevalence rates and to consider the potential regional differences throughout Japan.

## Material and methods

### Study design

The Japanese Society of Pediatric Dentistry requested the cooperation of 460 private dental clinics or university hospitals, which specialize in pediatric dentistry, at district prefecture facilities throughout Japan.

The subjects consisted of about 5000 Japanese children aged 7–9 years across Japan. The study aimed to include approximately 1/3000 of the child population in each prefecture of Japan. Subjects were randomly selected in each clinic or university hospital.

Finally, informed consent was obtained from 4985 children or the children’s guardians. Inclusion criteria included children aged 7–9 years with no systemic disease history and four fully erupted permanent first molars. We selected the age group of 7–9 years as all four first permanent molars and incisors would have erupted in the oral cavity of children at this age, and children older than 9 years have a higher risk of caries development. Exclusion criteria were children with enamel hypoplasia or amelogenesis imperfecta and under orthodontic treatment at the assessment. This study was carried out between October 2015 and February 2016.

MIH prevalence rates throughout Japan were investigated by evaluating eight regions by the jurisdiction of the Japanese Ministry of Health, Labour, and Welfare based on 47 prefectures. A child’s residence was defined as the mother’s residence during pregnancy. The eight areas classified according to each prefecture are as follows:

i. Hokkaido (Hokkaido prefecture)
ii. Tohoku (Aomori, Iwate, Miyagi, Akita, Yamagata, and Fukushima prefectures)
iii. Kanto Shin-Etsu (Ibaraki, Tochigi, Gunma, Saitama, Chiba, Tokyo, Kanagawa, Niigata, Yamanashi, and Nagano prefectures)
iv. Tokai Hokuriku (Toyama, Ishikawa, Gifu, Shizuoka, Aichi, and Mie prefectures)
v. Kinki (Fukui, Shiga, Kyoto, Osaka, Hyogo, Nara, and Wakayama prefectures)
vi. Chugoku (Tottori, Shimane, Okayama, Hiroshima, and Yamaguchi prefectures)
vii. Shikoku (Tokushima, Kagawa, Ehime, and Kochi prefectures)
viii. Kyushu (Fukuoka, Saga, Nagasaki, Kumamoto, Oita, Miyazaki, Kagoshima, and Okinawa prefectures).

**Fig. 1:**
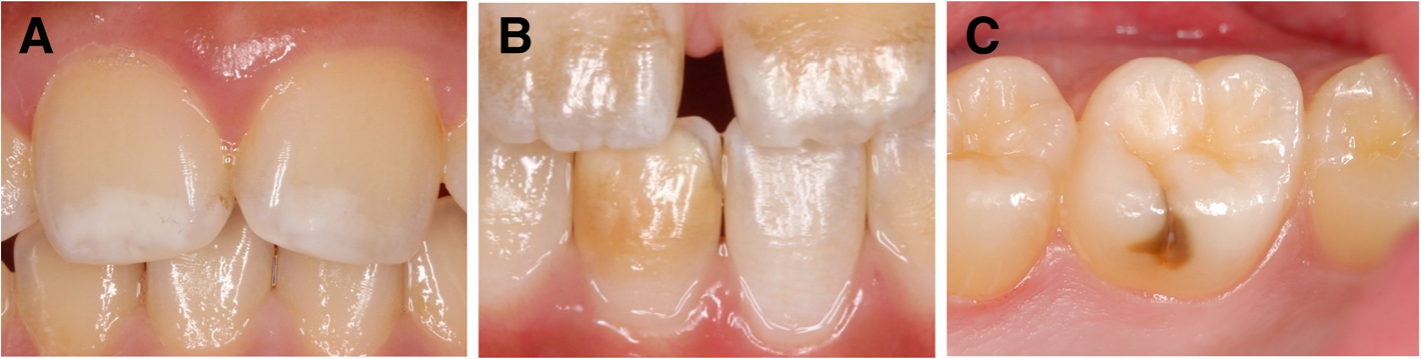
Tooth discoloration in a child with MIH was graded according to the color shade as follows: **a** white opacity, **b** yellow/brown opacity, and **c** dark brown opacity

**Fig. 2:**
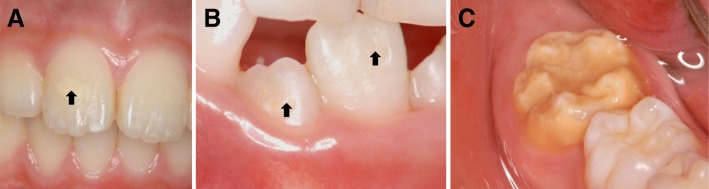
Tooth surface defects were categorized as follows: **a** none, **b** enamel defect, and **c** dentin defect

For calibration, the criteria index with 35 clinical photographs was prepared. The record chart guided by the criteria index was used in this study. The examiners in each area received lectures on how to use the criteria index. MIH was evaluated using criteria based on the 2003 European Academy of Pediatric Dentistry guidelines [10], with some modification. Briefly, tooth discoloration of a child with MIH was graded according to the color shade as white opacity, yellow/brown opacity, or dark brown opacity (Fig. 1). Tooth surface defects were categorized as none, enamel defect, or dentin defect (Fig. 2). The localization of demarcated opacities and enamel breakdown were recorded on a standard cord form using a guided record chart.

### Statistical analyses

The chi-square test was used to evaluate the differences in MIH prevalence rates between age groups, sex, and regions. Multivariate logistic regression analysis was used to evaluate whether regional differences in MIH prevalence exist. Age and sex were simultaneously entered in the multivariate model because age and sex are known to be determinants of MIH [1] and may be potential confounders for the association between regions and MIH. This analysis was set up to calculate the age- and sex-adjusted odds ratios (ORs) of age, sex, and regions for MIH, and their 95% confidence intervals (95%CIs). The multivariate models were examined using the Hosmer-Lemeshow goodness-of-fit test [11]. All statistical analyses were performed using SPSS 20.0 (SPSS Inc., Chicago, IL, USA). *p* values < 0.05 were considered statistically significant.

## Results

Approximately 84.3% (388/460) of private dental clinics or university hospitals throughout Japan agreed to cooperate with the study. A total of 4985 children were invited to participate. Of these, 489 children had incomplete data. Thus, 4496 (90.2%) children were included in the analyses. There was no significant difference according to sex (boys—2216, 49.3%; girls—2280, 50.7%). The overall prevalence of MIH in Japan was 19.8% (892/4496). Tooth surface defects were found in 8.1% (366/4496) of subjects. A summary of subjects’ characteristics is shown in Table 1.

**Table 1:** Distribution of MIH in areas throughout Japan and age- or sex-specific prevalence of MIH

	Number of subjects	Percent	Number of subjects with MIH	Prevalence (%)^§^	*χ*^2^ test	Odds ratio^a^
						(95% confidence interval)
Region						
Hokkaido	235	5.2	33	14.01 (13.97–14.06)	< 0.001	1.00
Tohoku	316	7.0	37	11.69 (11.65–11.72)		0.81 (0.49–1.34)
Kanto Shin-Etsu	1561	34.7	289	18.48 (18.46–18.50)		1.40 (0.95–2.07)
Tokai Hokuriku	601	13.4	116	19.26 (19.23–19.29)		1.48 (0.97–2.24)
Kinki	802	17.8	179	22.27 (22.25–22.30)		1.77 (1.18–2.65)
Chugoku	262	5.8	52	19.81 (19.76–19.86)		1.54 (0.95–2.48)
Shikoku	153	3.4	43	28.05 (27.98–28.12)		2.43 (1.46–4.04)
Kyushu	566	12.6	143	25.21 (25.20–25.23)		2.07 (1.37–3.13)
Age (years)						
7	1533	34.1	341	22.2	< 0.01	1.40 (1.16–1.68)
8	1631	36.3	324	19.9		1.21 (1.00–1.46)
9	1332	29.6	227	17.0		1.00
Sex						
Male	2216	49.3	428	19.3	0.38	1.00
Female	2280	50.7	464	20.4		1.07 (0.92–1.24)
Total	4496		892	19.8		

Hosmer-Lemeshow goodness-of-fit test, 0.11

^a^Odds ratios were adjusted for sex and age

^§^Prevalence by region was adjusted for sex and age

**Fig. 3:**
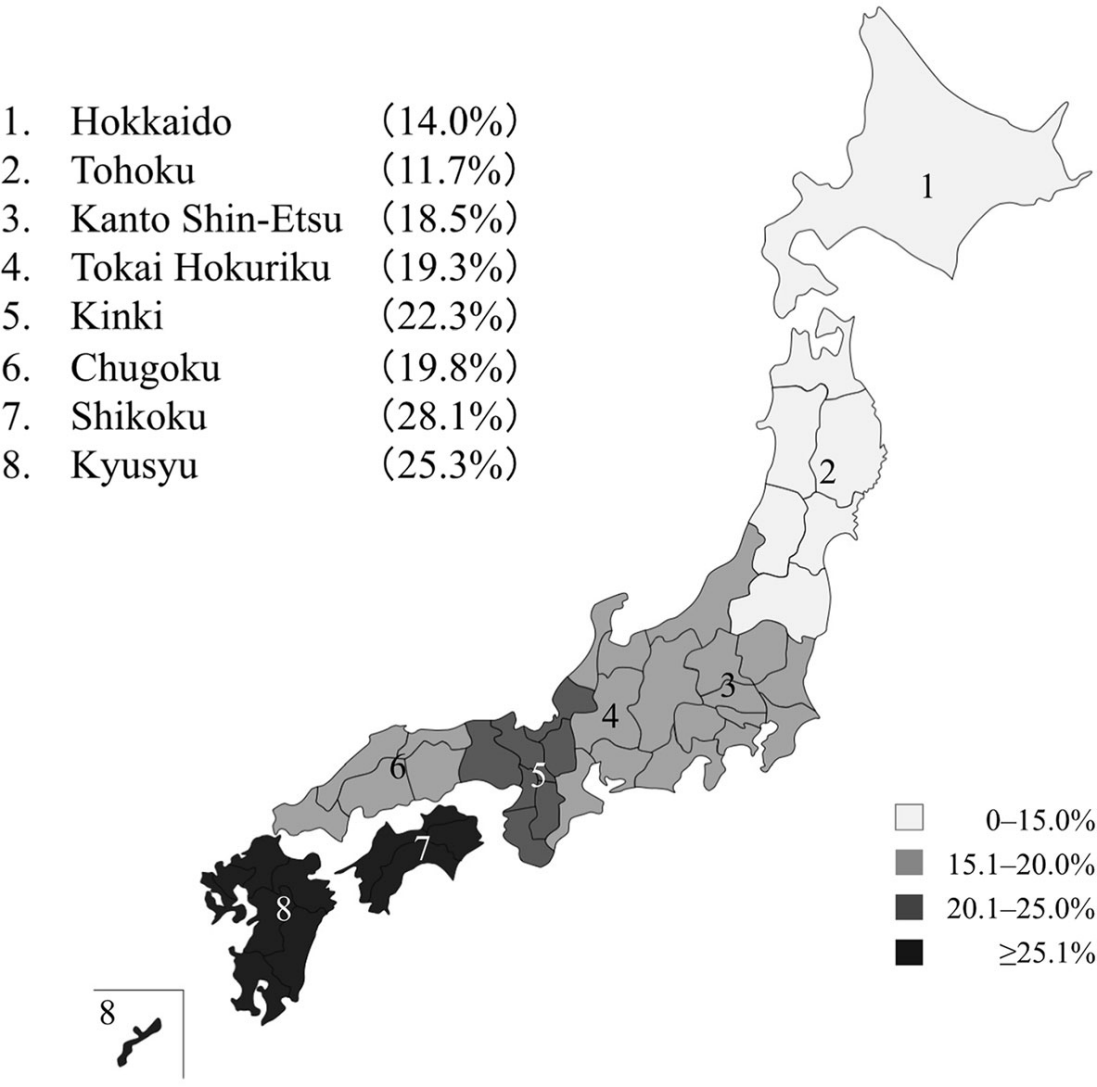
MIH prevalence rates throughout Japan. MIH exhibited low prevalence in northeastern areas and high prevalence in southwestern areas of Japan

The distribution of MIH in the eight classified areas throughout Japan is shown in Table 1 and Fig. 3. The MIH prevalence rates were 14.0% in Hokkaido, 11.7% in Tohoku, 18.5% in Kanto Shin-Etsu, 19.3% in Tokai Hokuriku, 22.3% in Kinki, 19.8% in Chugoku, 28.1% in Shikoku, and 25.3% in Kyushu. MIH exhibited low prevalence in northeastern areas and high prevalence in southwestern areas of Japan (Fig. 3). MIH prevalence rates throughout Japan showed a similar tendency in the multivariate logistic regression analysis. The adjusted ORs for MIH with Hokkaido as the reference group were significantly high in Kinki (OR 1.77, 95% CI 1.18–2.65), Shikoku (OR 2.43, 95% CI 1.46–4.04), and Kyushu (OR 2.07, 95% CI 1.29–3.20). Furthermore, MIH frequency significantly decreased with increasing age—22.2% in 7-year-olds, 19.9% in 8-year-olds, and 17.0% in 9-year-olds (*p* < 0.001). No significant sex difference in MIH prevalence was demonstrated (boys 19.3%, girls 20.4%).

## Discussion

This study clarified the prevalence of MIH among children aged 7–9 years and showed regional differences in MIH prevalence throughout Japan.

The overall prevalence of MIH in this study was 19.8%, which is similar to the rates reported in Sweden (18.4%) [12], Iraq (18.6%) [13], Finland (19.3%) [14], and Brazil (19.9%) [15]. This similarity in MIH prevalence suggests that MIH is a common clinical finding among children worldwide. The loss of enamel from permanent teeth in children is a frequent occurrence. MIH is associated with many dental problems, such as hypersensitivity, esthetics, and rapid dental caries progression [16]. Therefore, clinical management of MIH is essential in the field of pediatric dentistry.

MIH was more prevalent among participants in southwestern areas (Kinki, Chugoku, Shikoku, and Kyushu) than those in northeastern areas (Hokkaido and Tohoku) of Japan. These regional differences could generate hypotheses for the analysis of the etiology of MIH in Japan. Many potential causes of MIH have been proposed, such as prematurity, viral or bacterial infections, respiratory diseases, asthma, and frequent episodes of fever in early childhood or mothers who experienced problems during pregnancy [7, 17]. However, these possible causes do not explain the regional differences in Japan. One potential explanation may be serum 25-hydroxyvitamin D concentrations in children or their mothers. Osteoporosis also occurs more frequently in southwestern areas than northeastern areas of Japan [18]. Patients with osteoporosis have low serum 25-hydroxyvitamin D concentrations [19]. Increases in serum 25-hydroxyvitamin D concentrations are significantly associated with a lower odds ratio of having MIH. Furthermore, higher serum 25-hydroxyvitamin D levels are related to a lower number of caries-affected permanent teeth [20]. Endogenous vitamin D3 is synthesized in the skin through exposure to ultraviolet B radiation from sunlight, and exogenous vitamin D3 is obtained from dietary sources including fatty fish, fish liver oil, egg yolks, and mushrooms [21, 22]. Vitamin D obtained from diet or ultraviolet B radiation undergoes hydroxylation in the liver, producing 25-hydroxyvitamin D, which is the active form of vitamin D [23]. Thus, serum 25-hydroxyvitamin D concentrations may vary according to the differences in eating habits, daylight hours, and outside time by area [24]. Japan is a very long country from the northeast to southwest direction. Since the daylight hours are longer and ultraviolet B radiation is greater in the southwestern part than the northeastern part of Japan, the prevalence of MIH may depend on the factors other than endogenous vitamin D3. Yaegashi et al. suggest that vitamin D intake, mainly from fish and fish products, is much higher in eastern areas of Hokkaido and Tohoku than in western areas of Japan. Thus, vitamin D intake itself, more than day length, might explain the lower incidence of hip fracture by osteoporosis [25]. Nutrient intake from fish might explain the regional differences we observed in the prevalence of MIH in Japan.

In this study, the prevalence of MIH decreased with increasing age. Hypomineralized teeth are prone to dental caries over time [26]. Although enamel hypomineralization would be masked by dental caries, the possibility of annual increases in MIH prevalence rates cannot be denied.

Our study has limitations. First, this study is not true random sampling. MIH has a difficult differential diagnosis from early caries, enamel hypoplasia after trauma, or amelogenesis imperfecta. Therefore, a dedicated specialist would be required to examine over 370 local clinics throughout Japan. Second, we did not evaluate socioeconomic conditions. However, almost all prefectures in Japan guarantee medical expenses for children. Since poverty rates, working poor rates, and child poverty rates in Kansai (and further west) and Tohoku (and further north) tend to be high [27], random sampling from each area (community-based design) would be ideal for the study. However, such a study is not feasible when considering the necessity of cooperation from local communities/schools throughout Japan. Therefore, we selected a clinic-/hospital-based study design. True MIH may be slightly lower than this study, but MIH is found by the dentist of the clinic. It is thought that this study reflects the actual situation.

## Conclusions

The overall prevalence of MIH in Japan was 19.8%. Regional differences in MIH prevalence rates were observed throughout Japan, with MIH occurring more frequently in southwestern areas than northeastern areas. To our knowledge, this is the first MIH study carried out in several regions throughout Japan.

## Abbreviation

MIH: Molar incisor hypomineralization
